# Cell-secreted Flavins Bound to Membrane Cytochromes Dictate Electron Transfer Reactions to Surfaces with Diverse Charge and pH

**DOI:** 10.1038/srep05628

**Published:** 2014-07-11

**Authors:** Akihiro Okamoto, Shafeer Kalathil, Xiao Deng, Kazuhito Hashimoto, Ryuhei Nakamura, Kenneth H. Nealson

**Affiliations:** 1 Departments of Earth Sciences and Biological Sciences, University of Southern California, Los Angeles, CA 90089; 2 Department of Applied Chemistry, University of Tokyo, Bunkyo-ku, Tokyo, 113-8654; 3 Biofunctional Catalyst Research Team, RIKEN Center for Sustainable Resource Science, Wako, Saitama 351-0198, Japan; 4 J. Craig Venter Institute, San Diego, CA 92121

## Abstract

The variety of solid surfaces to and from which microbes can deliver electrons by extracellular electron transport (EET) processes via outer-membrane *c*-type cytochromes (OM *c*-Cyts) expands the importance of microbial respiration in natural environments and industrial applications. Here, we demonstrate that the bifurcated EET pathway of OM *c*-Cyts sustains the diversity of the EET surface in *Shewanella oneidensis* MR-1 via specific binding with cell-secreted flavin mononucleotide (FMN) and riboflavin (RF). Microbial current production and whole-cell differential pulse voltammetry revealed that RF and FMN enhance EET as bound cofactors in a similar manner. Conversely, FMN and RF were clearly differentiated in the EET enhancement by gene-deletion of OM *c*-Cyts and the dependency of the electrode potential and pH. These results indicate that RF and FMN have specific binding sites in OM *c*-Cyts and highlight the potential roles of these flavin-cytochrome complexes in controlling the rate of electron transfer to surfaces with diverse potential and pH.

Extracellular electron transport (EET) is the process through which living microorganisms exchange electrons with the surface of extracellular insoluble substrates^1^,^2^,^3^. This interfacial electron transport process originally gained considerable attention within the realm of biogeochemical mineral cycling^1^. However, the known variety of surfaces with which the cells can exchange electrons has since expanded the significance of EET in areas such as iron-corrosion^4^, microbial fuel cell research^3^,^5^, soil remediation^6^ and bioelectrochemistry^7^,^8^. Nonetheless, the mechanism by which multiple EET-related proteins integrated at the cell outer-membrane (OM) accomplish electronic connections with such diverse solid subsurface conditions remains difficult to ascertain. *Shewanella* *oneidensis* MR-1 and *Geobacter sulfurreducens* are capable of performing EET to a number of different solid-phase electron acceptors via OM *c*-type cytochromes (*c*-Cyts) having multi-heme redox centers^9^,^10^. Although purified OM *c*-Cyts from these microbes are well characterized^11^,^12^, their roles in the whole protein complex *in-vivo* remain poorly understood. Thus, the role of complex formation by multiple OM *c*-Cyts and the mechanisms by which they work together for interfacial electron delivery to diverse insoluble substrates are subjects of ongoing debate^13^,^14^,^15^.

In addition to the heme redox centers in OM *c*-Cyts, cell-secreted flavin molecules such as riboflavin (RF) and flavin mononucleotide (FMN) (Fig. 1a) appear to be the major electron carriers that terminate EET processes at the interface between cells and electrodes in both *S. oneidensis* MR-1^16^,^17^ and *G. sulfurreducens*^18^,^20^. In *S. oneidensis* MR-1, these flavin compounds deliver electrons from OM *c*-Cyts, MtrC and OmcA proteins to extracellular insoluble substrates^16^,^17^. As a number of works have shown that MR-1 can use soluble redox substrates to reduce extracellular solids^19^,^20^,^21^,^22^, early studies proposed that FMN and/or RF serve as diffusing electron shuttles between the OM *c*-Cyts and the electron acceptors via a two-electron redox reaction of free flavins (oxidized form 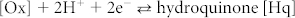)(Fig. 1b and d)^16^. As both RF and FMN were able to enhance the rate of EET to a similar degree, the two flavins were presumed to have the same function and reaction schemes for the EET processes^16^,^24^.

In contrast, we recently demonstrated that FMN acts as a cofactor bound to the MtrC protein under conditions of respiratory electron flow, forming a semiquinone (Sq) and accelerating the reaction via a one-electron reaction 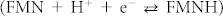 (Figs. 1c and e)^19^,^25^. This experiment utilized differential pulse voltammetry (DPV) of intact cells to monitor the redox reaction of FMN in a monolayer biofilm of MR-1. Compared with the two-electron redox reaction of free-form FMN, the observed peak potential (*E_p_*) of FMN in the differential pulse (DP) voltammogram shifted from −260 mV to −145 mV, and the half-width potential (Δ*E_p/2_*) was altered from 60 mV to 130 mV, indicating that the number of electrons involved in the reaction (*n*) was one. Together with electron paramagnetic resonance (EPR) detection of the free radical species in Sq, FMN was confirmed to mediate the redox reaction of Ox/Sq at the interface between the cells and electrodes (Fig. 1c). This alteration in the redox reaction provided much more thermodynamically favorable electron transfer kinetics than did the two-electron process of free-form FMN in the shuttling mechanism, demonstrating that the one-electron reaction of FMN provides a major pathway for flavin-mediated EET processes. Importantly, this alteration in the redox signal was not observed in the absence of electron source or in a mutant lacking the MtrC protein, which has been reported to have a possible flavin-binding site^7^,^26^, indicating that FMN enhances the EET process as a cofactor in MtrC protein with reduced hemes (Fig. 1e).

Flavins, including flavin adenine dinucleotide (FAD), FMN and RF, are known to be electron transport cofactors in virtually all organisms^27^,^28^,^29^ and generally retain their specific function and binding motif in known microbial enzymes^30^,^31^. For example, macrophage nitrogen monoxide synthases in eukaryotes are known to possess FAD and FMN cofactors for different locations and functions in their protein scaffolds^30^. Furthermore, in an integral membrane protein Na^+^ pumping NADH quinone oxidoreductase complex, FMN and RF (located on different subunits) cooperatively transport electrons to generate a sodium gradient^31^. Thus, it is possible that FMN and RF in *S. oneidensis* MR-1 differentially function at separate locations in OM proteins as cofactors, meaning that the OmcA protein associates with RF. In support of this concept, detailed crystal structural analysis^32^ and small-angle X-ray spectroscopy measurements with purified OmcA protein^33^ suggest that the OmcA protein has a potential flavin-binding site in the protein scaffolds. Therefore, it is plausible that the RF binds to OmcA protein scaffolds (Fig. 1e) and that the two flavin-binding OM *c*-Cyts might function differently for EET processes.

Because the flavin-binding site strongly alters the redox potential (*E_0_*) of flavin by its non-polarity and *π*-*π* stacking interactions^27^,^28^,^29^,^34^, the *E_0_* of flavin molecules serves as a good indicator for the interaction between flavin and its binding site. In this study, whole-cell DPV measurements with MR-1 cells were employed to detect RF binding of the OmcA protein. Mutant strains lacking the ability to produce either MtrC (Δ*mtrC*) or OmcA protein (Δ*omcA*) were used to confirm the interaction between flavins and OM *c*-Cyts. Furthermore, the dependency of the pH and electrode potential of the EET process via RF or FMN was examined to determine the difference in their electrode surface affinities.

## Results

### Comparison between the redox signals of RF and FMN during *in-vivo* current production in *S. oneidensis* MR-1

To investigate the redox state of the RF responsible for activating EET processes in *S. oneidensis* MR-1 cells, we conducted DPV measurements followed by the electrochemical cultivation of MR-1 grown on an ITO electrode in the presence of either RF or FMN. A cell suspension of MR-1 with an optical density of 0.1 at λ = 600 nm (OD_600_) was inoculated onto an ITO electrode poised at +200 mV (vs. Ag/AgCl KCl sat.) in the presence of either 4.0 μM RF or FMN and with 10 mM lactate as an electron source, as this electrode potential is positive enough for MR-1 cells to use electrodes as an electron acceptor^25^.

In the presence of RF, we observed comparable current productions of microbial lactate oxidation (approximately 15 μA/cm^2^) with FMN, and DPVs were measured before the current production was saturated (Supplementary Fig. S1a). As shown in Figure S1b, the baseline-subtracted DP voltammogram contained the redox signals of RF, yielding an *E_p_* at −110 mV (blue line in Fig. 2). The Δ*E_p/2_* of the RF peak was more than double the width than that of the free RF peak mediating the two-electron redox reaction (dotted line in Fig. 2), indicating that RF with MR-1 cells mediates a one-electron redox reaction^35^. These *E_p_* and Δ*E_p/2_* values of the RF with the cells are consistent with those of FMN, which was previously demonstrated to mediate a one-electron Sq/Ox redox reaction as a redox cofactor in the MtrC protein^25^. Moreover, the peak current of RF at *E_p_* = −110 mV in the DP voltammograms exhibited a positive correlation with the metabolic current production, as observed for FMN (Supplementary Fig. S3). These results indicate that RF enhances the rate of the EET process via Sq formation but that its redox profile is clearly different from that of FMN; in contrast, the redox profiles for free RF and FMN without microbes were almost identical (Supplementary Fig. S2).

### Effect of *OmcA* gene deletion on the current production and redox profile of bound RF

To examine our idea that RF associates with the OmcA protein as a redox cofactor to accelerate EET, we measured the current production and DPV of an *omcA* deletion mutant that is unable to produce the OmcA protein (Δ*omcA*). As shown in Figure 3a (blue and green lines), approximately the same extent of current generation in Δ*omcA* was observed with or without the addition of 2.0 μM RF. In contrast, the addition of FMN significantly enhanced the microbial current production of Δ*omcA* (Fig. 3a, black line), displaying a 10-fold current increase at t = 35 h compared with that in the absence of any added flavin (Fig. 3a, green line). The initial periods during which the current production remained at less-than hundred nA/cm^2^ in the systems was most likely due to the inability to attach to the electrode surface in comparison to the wild type (WT) because the purified OmcA protein functions by attaching to positively charged electrodes^36^. These results indicate that the RF does not accelerate the EET activity in the Δ*omcA* mutant, whereas FMN does, presumably by associating with the MtrC protein that is still present in this mutant.

Furthermore, in the presence of RF, the *E_p_* negatively shifted more than 80 mV compared to that of the WT and was located at an *E_p_* close to that of free RF (Fig. 3b). In contrast, the *E_p_* of FMN was observed at the almost identical *E_p_* of FMN for the WT strain (Fig. 3c). This significant effect of the *omcA* gene deletion on the redox profile of RF clearly demonstrated that the OmcA protein interacts with the RF to transport metabolically generated electrons. In addition, it is noteworthy that the EET process mediated by the MtrC protein of the Δ*omcA* mutant is enhanced by the presence of FMN. Furthermore, the redox profile of FMN in the Δ*omcA* mutant was similar to that observed in the WT. These results are consistent with the results of our previous study demonstrating that FMN specifically associates with the MtrC protein as a redox cofactor^25^.

### Effect of MtrC gene deletion on the current production and redox cycling of OmcA-bound RF

To confirm that RF associates with the OmcA protein and thereby enhances current production in *S. oneidensis* MR-1, we examined whether RF could enhance the EET activity of the OmcA protein remaining in a Δ*mtrC* mutant strain. As expected, an approximately 10-fold greater current was produced with the Δ*mtrC* strain in the presence of 2.0 μM RF (Fig. 4a, black line) than in the absence of RF (Fig. 4a, green line). Moreover, the DP voltammogram measured after saturation of the current production indicated *E_p_* and Δ*E_p/2_* values of −102 mV and 150 mV, respectively (Fig. 4b). This redox profile corresponded well to that of RF observed for the WT strain (Fig. 2), clearly demonstrating that RF enhances the EET activity in the Δ*mtrC* mutant but not in the Δ*omcA* strain. These data strongly support our conclusion that the OmcA protein is activated by the association of RF as a cofactor but not by the association of FMN. In addition, we also confirmed that the presence of 2.0 μM FMN caused a significantly smaller current enhancement and *E_p_* shift from free FMN in the DP voltammogram of the Δ*mtrC* mutant as compared with RF (Fig. 4c). This small current enhancement could be due to a portion of FMN which is spontaneously hydrolyzed to RF^37^ or the uptake of FMN by the homologous protein of MtrC, MtrF protein, synthesized in Δ*mtrC*^38^. However, the significant loss of ability of Δ*mtrC* (compared to WT) to enhance EET via the formation of Sq using FMN was clearly demonstrated; i.e., FMN can specifically function as a redox cofactor in the MtrC protein but not in the OmcA protein.

### Electrode potential and pH dependency for EET enhancement by RF and FMN

Because bound RF and FMN are both terminal electron carriers from OM *c*-Cyts to the electrode in the EET process (Supplementary Fig. S4), their corresponding bifurcated electron pathways should have different affinities to charged surfaces. To test this hypothesis, EET enhancement by the addition of RF or FMN was compared at several electrode potentials and pH values. The electrode potential dependency on the EET enhancement was measured in a monolayer biofilm of MR-1 at +0.4 V (vs. SHE) on the ITO electrode in the presence of lactate^39^. After the electrode potential alteration from +0.4 V to −0.2 V and from +0.4 V to +0.8 V, an enhancement factor for EET was calculated by comparing the microbial current production before and after the addition of 2 μM RF or FMN. Inertness of these flavins to the electrode at −0.2, +0.4 and +0.8 V was confirmed by electrochemical experiments in the absence of the cells in the electrochemical reactor (Fig. S5).

As shown in Figure 5a, the enhancement factor for both RF and FMN increased as the potential shifted in the negative direction, consistent with previous reports^40^. However, FMN exhibited a significantly higher enhancement factor (approximately 15-fold) compared with RF (approximately 3-fold) at an electrode potential of −0.2 V, whereas the difference between RF and FMN was less significant at 0.4 and 0.8 V. These results indicate that the FMN-MtrC complex is more suitable for transporting electrons to surfaces with a negative electrode potential as compared to the RF-OmcA complex. Subsequently, we tested the pH dependency of EET enhancement by RF and FMN at an electrode potential of +0.4 V. With a pH change from 6 to 9 in the electrolyte solution, the enhancement factor for RF and FMN remained largely unchanged (Fig. 5b). However, the pH dependency displayed a distinct difference between RF and FMN: the enhancement factor of FMN increased with increasing pH, whereas RF showed a minimum and maximum at pH 7 and 6, respectively (Fig. 5b). Checking the effect of ionic strength change associated with the pH control to 6 or 9 by the addition of neutral salt, little effect on the current production of MR-1 was observed (Fig. S6). Together, these data clearly indicate that each flavin has a different range of potential and unique pH dependency to compensate for the EET enhancement, suggesting that this EET pathway bifurcation contributes to sustaining the versatile use of varied charged surfaces for EET.

## Discussion

Protein purification and *in-vivo* cross-linking experiments have confirmed that the MtrC protein forms a complex with the OmcA protein in *S. oneidensi*s MR-1^13^,^14^. However, these two proteins have a similar enzymatic capacity for transporting electrons to the surface of electrodes and minerals in a purified protein system^15^, and thus the functional difference between OmcA and MtrC has been questioned. In this work, we studied the role of extracellular RF and FMN to differentiate the functions of OmcA and MtrC in *S. oneidensi*s MR-1 by whole-cell electrochemistry with WT and mutant strains lacking the MtrC or OmcA protein. A physiological concentration (2 μM) of RF was demonstrated to interact with the OM protein, OmcA, to produce a change in the redox potential of the RF from −260 mV to −110 mV, thereby generating an Sq intermediate with a more favorable redox potential for receiving electrons from hemes in the OmcA protein^25^,^41^.

**Figure 1:**
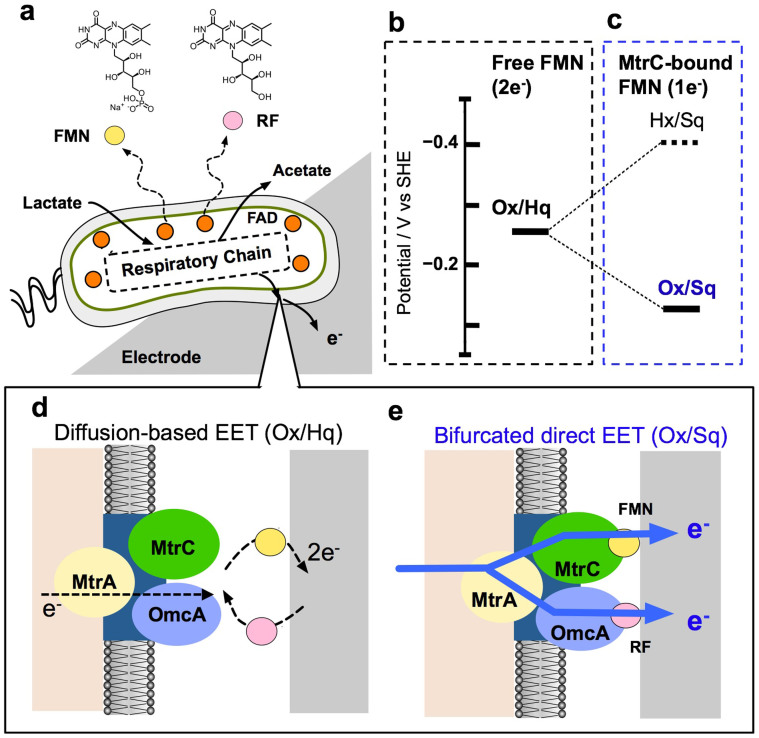
(a) Schematic illustration of the extracellular electron transfer process to the electrode in *S. oneidensis* MR-1 and flavin mononucleotide (FMN) and riboflavin (RF) secretion via enzymatic conversion of flavin adenine dinucleotide (FAD). Energy diagram depicting the two-electron and one-electron redox reactions of dissolved free FMN (b) and FMN bound to OM *c*-Cyts and MtrC protein (c), respectively. (d) Model representation of electron shuttling between the electrode surface and OM *c*-Cyts, MtrC and OmcA proteins via a two-electron redox reaction of free-form RF and FMN. (e) Illustration of the bifurcated direct electron transport process via one-electron redox cycling of FMN and RF bound to the MtrC and OmcA proteins, respectively, enhancing the electron delivery to the electrode surface.

**Figure 2:**
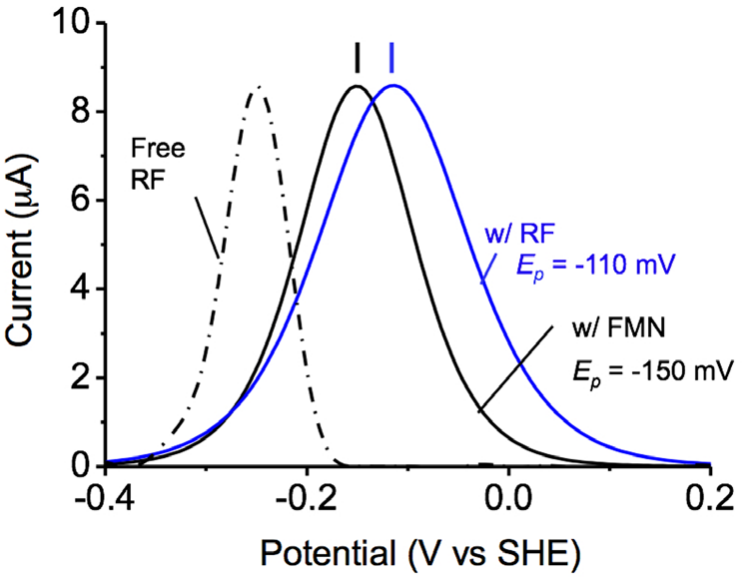
Differential pulse voltammogram for *S. oneidensis* MR-1 cells after 18 h of electrode cultivation at +0.4 V (vs. SHE) in the presence of 4.0 μM FMN (w/FMN, black line) and RF (w/RF, blue line). The dotted line depicts the data points for the free RF solution. The peaks of the blue and black lines were deconvoluted from raw data in Figure S1 b and c, and the peak currents were normalized. The peak potential of electrochemical signals was described as *E_p_*.

**Figure 3:**
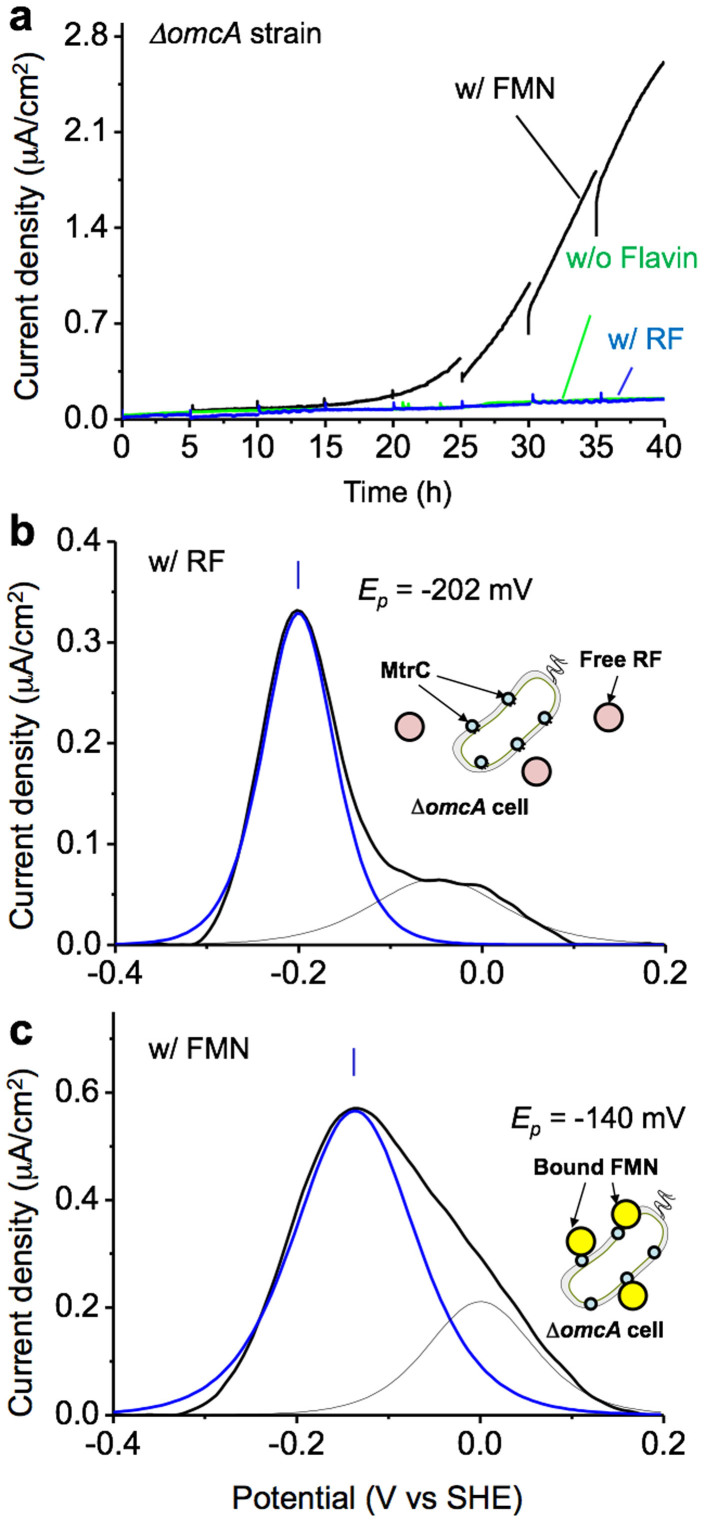
(a) Microbial current production versus time for Δ*omcA* cells of the *S. oneidensis* MR-1 strain inoculated in electrochemical cells with medium containing 10 mM lactate and either 2.0 μM FMN (w/FMN, black), 2.0 μM RF (w/RF, blue) or no addition of flavin (w/o Flavin, green). Base-line subtracted differential pulse voltammetry for Δ*omcA* cells conducted at t = 35 h in panel a in the presence of 2.0 μM RF (b) and FMN (c). In panels b and c, the blue and black lines depict the data points for the flavin and heme redox reactions, respectively, deconvoluted from raw data shown as the solid black line. The same trend was reproduced at least three times in separated experiments.

**Figure 4:**
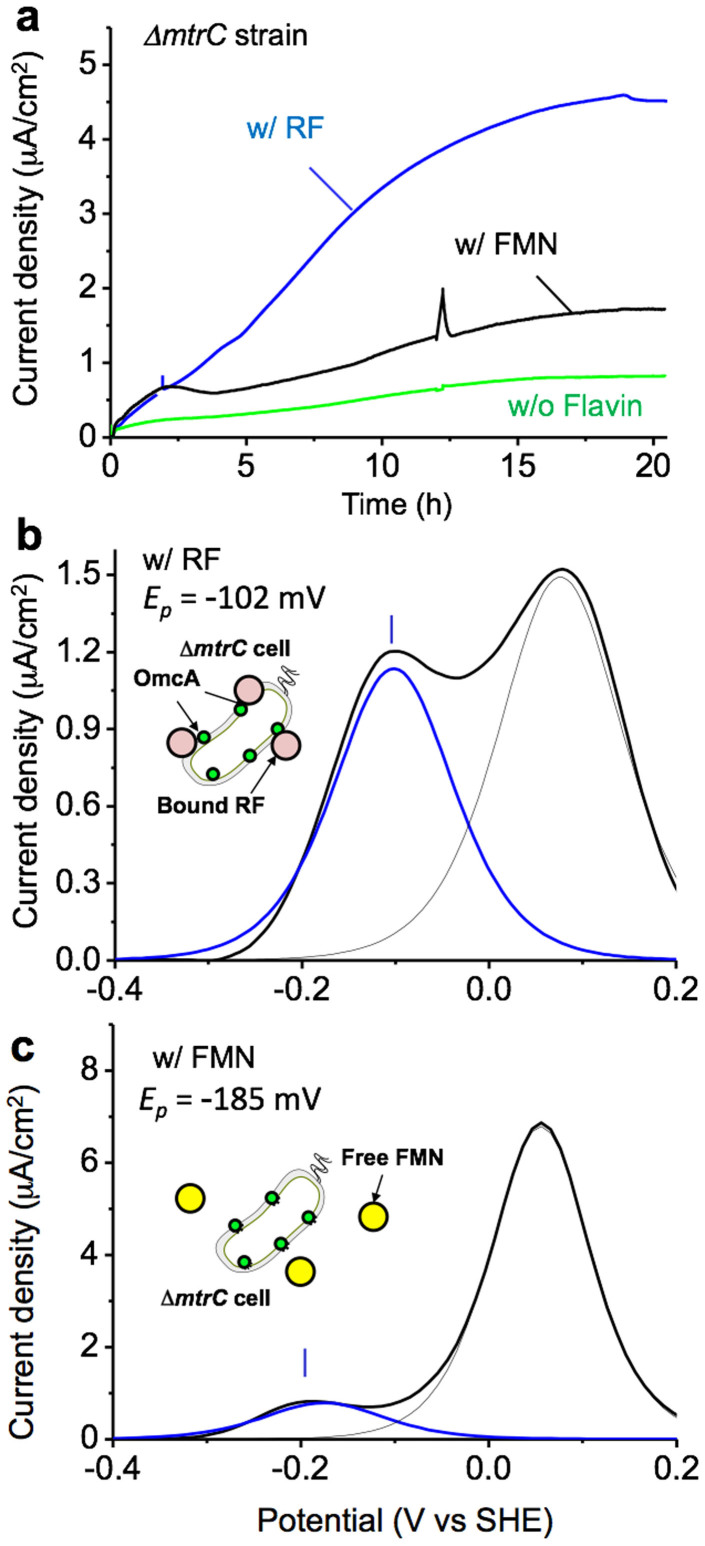
(a) Microbial current production versus time for Δ*mtrC* cells of the *S. oneidensis* MR-1 strain inoculated in electrochemical cells with medium containing 10 mM lactate and either 2.0 μM FMN (w/FMN, black), 2.0 μM RF (w/RF, blue) or no addition of flavin (w/o Flavin, green). Baseline-subtracted differential pulse voltammetry for Δ*omcA* cells conducted at t = 21 h in panel a in the presence of 2.0 μM RF (b) and FMN (c). In panels b and c, the blue and black lines depict the data points for the flavin and heme redox reactions, respectively, deconvoluted from raw data shown as the solid black line. The same trend was reproduced at least three times in separated experiments.

**Figure 5:**
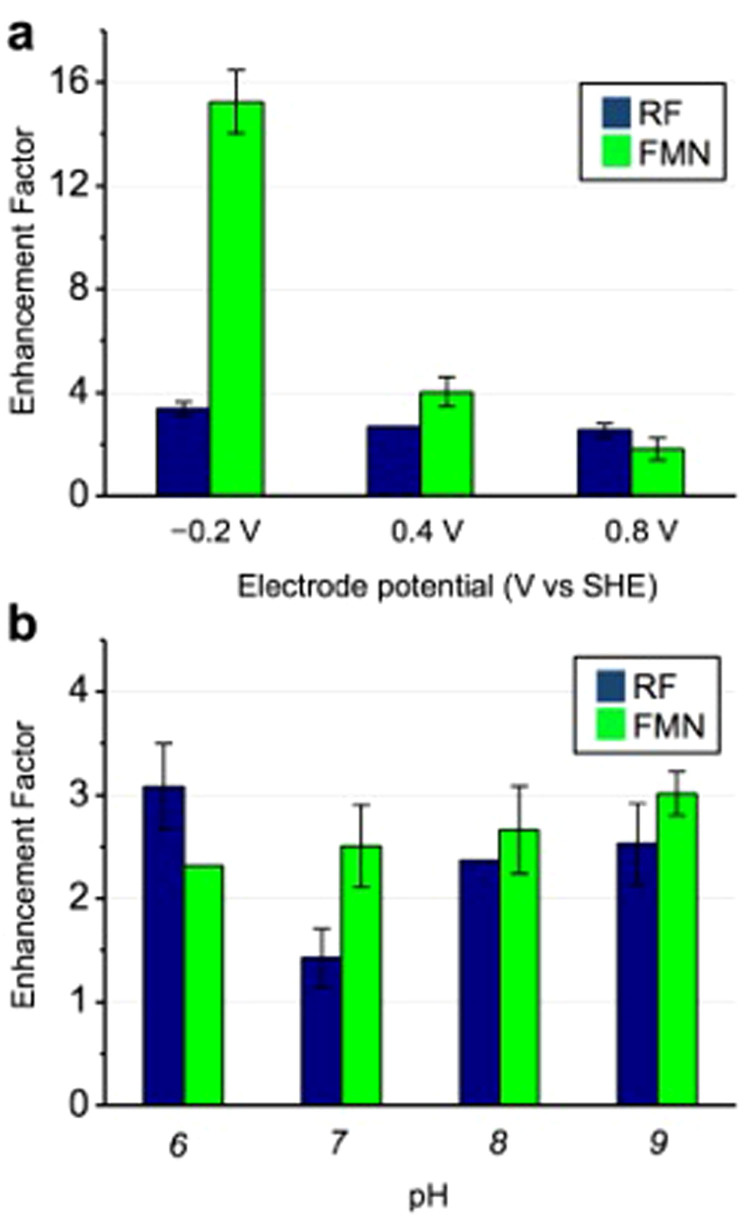
Electrode potential and pH dependency of enhancement factor in the microbial current production of *S. oneidensis* MR-1 determined by the addition of either 2 μM riboflavin (RF) or flavin mononucleotide (FMN). (a) The current production was compared before and after flavin addition at pH 7.8 at electrode potentials of −0.2, 0.4 and 0.8 V vs. SHE. (b) The current production was compared before and after flavin addition at an electrode potential of 0.4 V at pH 5.6, 7.3, 7.8 and 8.6. Error bars indicate the standard error of the mean values calculated with data obtained from three or more individual experiments.

A deletion mutant lacking the OmcA protein does not exhibit this positive shift in the RF redox potential, whereas a mutant lacking the MtrC does, indicating that RF specifically associates with OmcA but not with MtrC. In a complementary manner, a specific interaction between the FMN and MtrC protein scaffolds was confirmed to enhance the EET capability of the MtrC protein as a redox cofactor (Fig. 1e). The present data regarding the difference between RF and FMN at a low (μM) concentration are not consistent with the reported shuttling mechanism involving flavin molecule diffusion with two-electron redox cycling (Fig. 1d). However, these findings are in agreement with our assumption that extracellular FMN and RF enhance EET as redox cofactors in the OM *c*-Cyts in *S. oneidensi*s MR-1^25^,^41^ and demonstrate how flavins activate flavoproteins in a wide range of microorganisms^42^. Therefore, it is most plausible that two different OM *c*-Cyts use a specific flavin as a redox cofactor to accelerate the EET process at the interface between MR-1 and electrodes via the generation of Sq (Fig. 1e). In addition to the ability to bind different flavins, when the pH and electrode potential were altered, we observed differences between flavin-bound MtrC and OmcA proteins with respect to their EET ability to the electrode (Fig. 5). The EET enhancement factor presented in Figure 5 is an indicator of the efficiency of bound flavins to electronically bridge the electrode surfaces and heme redox centers in OM *c*-Cyts. The electrode potential dependency for the EET enhancement factor shown in Figure 5a highlights the feasibility of MtrC-bound FMN to transport electrons to a negative potential electrode (−0.2 V). A more negative redox potential of MtrC-bound FMN at an *E_p_* of −150 mV compared with OmcA-bound RF at −110 mV clearly led to enhanced EET kinetics to the electrode at −0.2 V (Fig. 2).

We also observed a large enhancement factor in the negative potential region, which was consistent with a previous report in which the detachment of OM *c*-Cyts from the electrode surface in *S. oneidensis* MR-1 caused a significant increase in EET enhancement by RF^40^. This finding implies that excess electrode binding of OM *c*-Cyts may impair the EET capability via the bound flavins. This insight is consistent with the pH dependency data showing that the OmcA-RF complex exhibited a minimum enhancement factor at pH 7 (Fig. 5b). The purified OmcA protein displayed the strongest affinity (at approximately pH 7) toward the surfaces of Fe_2_O_3_ and Al_2_O_3_^43^, which have an isopotential point similar to that of an ITO electrode. In contrast, a pH change from 6 to 11 did not affect the amount of purified MtrC protein adsorbed onto the electrode surface^44^.

These findings suggest that the high electrode affinity of OM *c*-Cyts could suppress the process of EET from bound flavin to the electrode surface. Combined with such interactions between OM *c*-Cyts and bound flavins, the observed dependency in EET enhancement indicates that the bound RF and FMN cover distinct ranges of local pH values and surface charges on the electrode surface. Our present experiments focused only on the electrode affinity, although it will be of great interest to examine the interaction of each bound flavin in OM *c*-Cyts using other known solid electron acceptors or conductors, e.g., Fe_2_O_3_, MnO_2_ and FeS^1^,^45^,^46^,^47^. Together with the specific binding affinity of flavins to the MtrC and OmcA proteins in *S. oneidensis* MR-1, this bifurcation of the EET pathway most likely expands the variety of surfaces to which MtrC or OmcA proteins can deliver electrons.

Although the specific affinity of flavins to OmcA and MtrC proteins was clearly demonstrated in our experiments, their putative flavin-binding domains have a high similarity in their overall tertiary structure^32^,^48^. As displayed in the amino acid sequence alignment of domain III in the OmcA and MtrC proteins (Fig S7), these two proteins share highly similar chemical characteristics according to criteria set in the T-Coffee program^49^ with the exception of approximately 10 amino acid insertions observed between residues 123–131 in OmcA and residues 133–147 in MtrC (Fig. S7).

In flavodoxin, which is a model FMN-binding protein that stabilizes the Sq state, the amino acid sequence alignment among various flavodoxins demonstrated that an intervening section of approximately 20 amino acid residues strongly increases the binding constant of RF to the apoflavodoxin lacking the FMN cofactor^50^,^51^. Thus we expect that a similar small insertion of amino acids in the alignment of both OmcA and MtrC proteins might provide the specific recognition of either FMN in MtrC or RF in OmcA. Conversely, it should be noted, in terms of the interaction between MtrC and OmcA proteins, that the specific flavin affinities of MtrC and OmcA were conserved even in the *ΔmtrC* and *ΔomcA* strains. This observation suggests that a protein-protein interaction between the OmcA and MtrC protein is not a major factor contributing to the interaction with flavins. This insight may provide the opportunity to examine how a similar flavin cofactor can have a distinct pH and electrode-potential dependency in the OmcA and MtrC protein scaffolds *in vitro*. Meanwhile, respiratory electron flow is important to stabilize the bound flavin cofactor as the Sq intermediate^25^, which supposedly prevents its direct detection in *in-vitro* studies. Flavin cofactors are not always stable in the protein pocket, i.e. some flavoproteins, when purified, have no flavin at their active sites^31^. Probably when respiratory electron flow takes place, reduced hemes in OM *c*-Cyts alter the protein structure to increase the flavin affinity^25^.

While the peak intensity of DP voltammogram is not quantitative, the concentration or binding dissociation constant of flavin bound to OM *c*-Cyts can be roughly estimated. The cell surface coverage of OM *c*-Cyts has been quantified as 10–30% by electrochemistry^52^ and antibody recognition force microscopy^53^. Assuming our electrode (surface area: 3.14 cm^2^) contains a monolayer biofilm and few planktonic microbes, the concentration of OM *c*-Cyts is several sub-nanomolar, and this should be the upper limit for the concentration of bound flavin in OM *c*-Cyts, because supposedly each MtrC or OmcA binds a single flavin as a cofactor in extracellular space^32^. The dissociation constant (K_d_) for the binding process between flavin and OM *c*-Cyts can be also quantified in some extent by analyzing the DPV signal of bound flavin in several different concentrations as shown previously^25^. Assuming, when we added 52 μM FMN to the monolayer biofilm of MR-1, OM *c*-Cyts is almost saturated with flavin cofactors, the peak current at *E_p_* of −145 mV was estimated approximately as 7 μAcm^−2^ by peak deconvolution analysis. As the K_d_ corresponds to the flavin concentration at which the binding site in OM *c*-Cyts is half occupied, the K_d_ should be around 10 μM. This value is a lot more than the physiological concentration of secreted flavin, indicating about 10% of OM *c*-Cyts is occupied by the flavin cofactor in the presence of 1.0 μM. The estimated K_d_ value is consistent with observations that the artificial addition of flavins causes an immediate current production increase in MR-1 cells and that the flavin species accumulate in the supernatant of MR-1 culture medium^16^,^17^. The K_d_ also indicates that the supernatant replacement with fresh medium in an electrode biofilm reactor immediately impairs current production at posed potential in *S. oneidensis* MR-1^16^, because it causes not only the reduction in soluble flavin concentration, but also significant loss of bound flavin cofactor. Thus, associated with the K_d_ value, the EET model based on the bound flavin cofactor fits with the reports of the effect of flavin on MR-1 cells. Meanwhile, only the shuttling model can explain the reported data for electron transport processes to iron in nano-porous glass beads^22^ or the electrode covered by an insulating layer with nano-scale holes^23^, where cells cannot make direct physical contact. In a shuttling process, it is hypothesized that free flavins receive respiratory electrons from hemes in the β-barrel domain of MtrC or OmcA protein, and diffuse to the electrode or mineral surface to donate electrons^26^. If the bound Sq occupies the binding site, probably the bound Sq or other hemes in OM *c*-Cyts are in charge of soluble free flavin reduction. The rates of RF and FMN reduction were reported to be almost equal in Δ*omcA* and Δ*mtrC* strain^54^: i.e., that soluble flavins are not reduced by a bound Sq but by heme exposed to exterior reduce soluble flavin. Meanwhile the flavin concentrations used in these experiments were quite high (120 μM), which may account for no selectivity for RF or FMN. However, as more is learned about the structure(s) of the Mtr proteins, as binding sites are identified^32^, and as experiments are done with pure proteins and at physiologically relevant flavin concentrations, these issues will likely be resolved.

In conclusion, we provide evidence that RF and FMN bound to OM *c*-Cyts bifurcate the EET pathway and sustain the variety of surfaces available for EET. This functional difference in each OM *c*-Cyt by specific flavin binding should be conserved in other EET-microbes, such as *G. sulfurreducens*^18^, and is connected with their survival capability in various subsurface environments. Further understanding of molecular-level interactions between flavins and their binding motifs in OM c-Cyts may provide us with a strategy to control the rate of EET, which could be important for versatile EET-related applications including microbial fuel cells^3^,^5^, microbial corrosion protection^55^, and bioremediation^6^.

## Methods

### Strains and culture conditions

*S. oneidensis* MR-1 was grown aerobically in 2.0 mL Luria-Bertani (LB) medium (20 g L^−1^) at 30°C for 20 h. The culture was then centrifuged at 6,000 × *g* for 10 min, and the resultant cell pellets were resuspended in 2.0 mL of defined medium (DM; NaHCO_3_ [2.5 g], CaCl_2_·2H_2_O [0.08 g], NH_4_Cl [1.0 g], MgCl_2_·6H_2_O [0.2 g], NaCl [10 g], and (2-[4-(2-hydroxyethyl)-1-piperazinyl] ethanesulfonic acid [HEPES; 7.2 g], yeast extract [0.5 g] [per liter])^56^ supplemented with 10 mM lactate as the sole carbon source (DM-L). The cells were further cultivated aerobically at 30°C for two nights, centrifuged for 10 min, and the resultant cell pellet was washed with DM prior to being used for electrochemical experiments. Mutant strains deficient in the genes encoding either *mtrC* were previously constructed by allele replacement using a two-step homologous recombination method^57^. The mutant strain lacking the OmcA protein (*ΔomcA*) was kindly supplied by Jim Fredrickson at the Pacific Northwest National Laboratory.

### Electrochemical measurements

A single-chamber, three-electrode system for whole-cell electrochemistry was constructed as described previously^46^. A tin-doped In_2_O_3_ (ITO) substrate (surface area: 3.1 cm^2^) placed at the bottom of the reactor was used as the working electrode, and Ag/AgCl (sat. KCl) and a platinum wire (approximate surface area: 10 mm^2^) were used as the reference and counter electrodes, respectively. Five milliliters of DM-L with certain concentration of RF (Wako Pure Chemical Industries, regent grade, >97%) or FMN (Wako Pure Chemical Industries, regent grade, >92%) was added into the electrochemical cell as an electrolyte and was deaerated by bubbling with N_2_ for more than 30 min. Oxygen concentration was monitored using a Microx TX3 trace instrument (PreSens, Munich, Germany) and it was maintained lower than 0.1 ppm. Cell suspensions with an optical density of 0.1 at 600 nm (OD_600_) were inoculated into the reactor with the electrode poised at a potential of +0.2 V (vs. Ag/AgCl KCl sat.). The reactor temperature was maintained at 30°C with no agitation during the measurements. DPV was conducted with an automatic polarization system (Reference600; GAMRY Instruments, Pennsylvania, USA) using 5.0 mV pulse increments, a 50 mV pulse amplitude, a 300 ms pulse width and a 5.0 s pulse period. Measurements were performed for sampling for 10 ms after each pulse. We used SOAS software, which is an open source program to analyze experimental electrochemical data^56^. The background current was subtracted by fitting the baseline from regions sufficiently far from the peak assuming continuation of a similar and smooth charging current throughout the peak region^58^. The background-subtracted data were further analyzed for deconvolution to fit the shapes of the peaks with the functions of a model function by finding the parameters that minimized the sum of the square weighted orthogonal distances from a set of observations to a curve determined by the parameters^58^. To calculate the EET enhancement factor for the 2 μM flavin addition in Figure 5, the microbial current production of MR-1 by lactate oxidation at −0.2, +0.4 and +0.8 V was measured immediately before and 1000 s after the addition in the presence of a monolayer biofilm and 10 mM lactate. The background current at electrode potentials of −0.2, +0.4 and +0.8 V was measured with the monolayer biofilm in the absence of lactate. In addition, the pH dependency of RF and FMN on EET was examined by changing the pH from 6 to 9 and by adding either H_2_SO_4_ or NaOH solutions prior to the electrochemical cultivation at +0.4 V. The effect of ionic strength was examined by the addition of NaCl to an electrolyte medium we used for current production measurements.

## Author Contributions

A.O. and K.H.N. designed the study. A.O., S.K. and X.D. conducted the research. A.O., R.N., K.H. and K.H.N. wrote the paper.

## Supplementary Material

Supplementary Information Figure S1, S2, S3, S4, S5, S6, and S7
