# Supplementary material for: Cell-secreted Flavins Bound to Membrane Cytochromes Dictate Electron Transfer Reactions to Surfaces with Diverse Charge and pH
Source: Sci Rep. 2014 Jul 11;4:5628. doi: 10.1038/srep05628 (PMC4092373; doi:10.1038/srep05628)
Supplement: Supplementary Information — Figure S1, S2, S3, S4, S5, S6, and S7 [file srep05628-s1.doc]

**Title: Cell-secreted Flavins Bound to Membrane Cytochromes Dictate Electron Transfer Reactions with a Diversity of Charged Surfaces**

Akihiro Okamoto^1,2^, Shafeer Kalathil^2^, Xiao Deng^2^, Kazuhito Hashimoto^2^, Ryuhei Nakamura^3^, Kenneth H. Nealson^1,4^*

*1. Departments of Earth Sciences and Biological Sciences, University of Southern California, Los Angeles, CA 90089. 2. Department of Applied Chemistry, University of Tokyo, Bunkyo-ku, Tokyo, 113-8654. 3. Biofunctional Catalyst Research Team, RIKEN Center for Sustainable Resource Science, Wako, Saitama 351-0198, Japan. 4. J. Craig Venter Institute, San Diego, CA 92121.*

E-mail: knealson@usc.edu

Supplementary Information

Fig. S1 (a) Microbial current production versus time for wild-type (WT) of *S. oneidensis* MR-1 strain inoculated in electrochemical cells with medium containing lactate 10 mM and either 4.0 μM FMN (black) or RF (blue). Differential pulse voltammetry for Δ*omcA* cells conducted at t = 18 h in panel (a), in the presence of 2.0 μM RF (b) and FMN (c). In panel b and c, blue and black lines depict the data points for flavin and heme redox reaction, respectively, deconvoluted from raw data in solid black line. The same trend was reproduced at least three times in separated experiments.

Fig. S2 Differential pulse voltammetry for 4.0 μM FMN and RF solution. The peak current were normalized to compare the redox profile.

Fig. S3 Plot of bacterial current production at an electrode potential of +200 mV (vs. Ag/AgCl KCl sat.) against the peak current of FMN (a) and RF (b) as determined by differential pulse voltammetry (DPV). The squares of the correlation coefficients were estimated by the addition of the point of origin to the obtained data. Microbial current production data was obtained before a DPV measurement.

Fig. S4. Schematic illustration of bifurcation in extracellular electron transport (EET) process in outer membrane *c*-type cytochromes (OM *c*-Cyts) complex in *S. oneidensis* MR1. Flavin mononucleotide (FMN) and riboflavin (RF) specifically associate with MtrC (a) and OmcA (b) protein, respectively, as a redox cofactor to transport electrons.

Fig. S5. Current versus time in the absence of microbial cells with medium containing 10 mM lactate under an aerobic condition at electrode potential of +0.8 (a, e), +0.4 (b, f) and -0.2 V vs SHE (c, g). Arrows indicate the time point 2 µM flavin mononucleotide (FMN) or riboflavin (RF) was added into the electrochemical reactor. Differential pulse voltammograms in the presence of 2 µM FMN (d) or RF (h) measured at 1200 s after posing electrode potential at +0.8 (black), +0.4 (blue) and -0.2 V vs SHE (red).

Fig. S6. Microbial current production versus time for wild-type (WT) of *S. oneidensis* MR-1 strain inoculated in electrochemical cells with medium containing lactate 10 mM with ion strength(*I*) 280 mM (black) and 300 mM (blue). The ionic strength was corrected by NaCl addition from 280 mM to 300 mM.

Fig. S7. Amino acid sequence alignment of domain III in MtrC and OmcA protein of *Shewanella oneidensis* MR-1. Alignment was generated using the T-Coffee program via the web site (http://tcoffee.crg.cat). Residues indicated by asterisks are conserved in all sequences. Residues indicated by single and double dots are of similar and highly similar chemical character respectively, according to criteria set in the program.
